# Relationship Functioning and Gut Microbiota Composition Among Older Adult Couples: Feasibility of Data Collection

**DOI:** 10.1093/geroni/igab046.2788

**Published:** 2021-12-17

**Authors:** Shelby Langer, M Aaron Guest, Melissa Tolson, Juan Maldonado Ortiz, John DiBaise, Helene Labonte, Rosa Krajmalnik-Brown

**Affiliations:** 1 Arizona State University The College of Nursing & Health Innovation, Phoenix, Arizona, United States; 2 Arizona State University, Phoenix, Arizona, United States; 3 Arizona State University, Tempe, Arizona, United States; 4 Mayo Clinic, Scottsdale, Arizona, United States

## Abstract

An emerging area of research extends work on couple functioning and physical health to gut health, a critical marker of general health and known to diminish with age. As a foray into this area, we conducted a pilot study to determine feasibility of data collection (questionnaires and a stool sample) among older adult couples. Participants were recruited from the community using a variety of methods including social media. Among 41 persons responding with interest across recruitment sources, 32 were contacted for screening. Inclusion criteria were: age 60+, marriage or cohabiting partnership, and English speaking/understanding. Exclusion criteria were a gastrointestinal disorder, receiving enteric nutrition, use of antibiotics (past month), cancer treatment (past 6 months), and a +COVID-19 diagnosis (past 2 months). Among 31 eligible couples, 30 consented. All 60 participants completed questionnaires and provided a stool sample using DNAgenotek’s OMR-200 collection kit, chosen for its ease and because samples can be stored at room temperature for 60 days. Sample characteristics were: M (SD) age = 66.57 (4.78); 53.3% female; 91.7% White; 1.7% Latinx; and 78.3% college-educated. 2 couples were same-sex. 43% reported at least one health condition and 25% reported use of a proton pump inhibitor (which can affect the gut microbiome), though none daily. Relational well-being was moderate-high on average per measures of dyadic adjustment and intimacy. Despite original plans to recruit couples in-person from a retirement community, remote operations were feasible via online assessment and study-coordinated shipping, a necessary yet fruitful shift due to the SARS-CoV-2 pandemic.

